# Machine Learning for Comparative Antidepressant Selection in Major Depressive Disorder: Systematic Review

**DOI:** 10.2196/89352

**Published:** 2026-05-13

**Authors:** Fiona He, Steven Huang, Richard Wang, Aland Chang, Jennifer L Phillips, Christopher Sun

**Affiliations:** 1 Digital Transformation and Innovation, Faculty of Engineering, University of Ottawa, Ottawa, ON, Canada; 2 Faculty of Science, University of British Columbia, Vancouver, BC, Canada; 3 Ottawa Heart Institute, Ottawa, ON, Canada; 4 Department of Physiology and Pharmacology, Western University, London, ON, Canada; 5 University of Ottawa Institute of Mental Health Research at The Royal, Ottawa, ON, Canada; 6 Telfer School of Management, University of Ottawa, 55 Laurier Ave E, Ottawa, ON, K1N 9B9, Canada, 1 (613) 562-5800 ext 4570

**Keywords:** machine learning, artificial intelligence, antidepressant, major depressive disorder, treatment selection, treatment outcome, precision psychiatry, personalized medicine

## Abstract

**Background:**

Major depressive disorder (MDD) affects approximately 1 in 6 adults during their lifetime, yet antidepressant selection relies predominantly on trial-and-error, with response rates of only 42% to 53%. While machine learning (ML) models have shown promise in predicting treatment outcomes, most focus on single treatments rather than comparative selection across therapeutic alternatives, limiting their clinical utility for the medication choice decisions that clinicians face in practice.

**Objective:**

This systematic review evaluates ML approaches that examine 2 or more pharmacological interventions for predicting treatment outcomes in MDD, with a focus on their capacity to facilitate comparative treatment selection between medications or medication classes for individual patients.

**Methods:**

Following PRISMA (Preferred Reporting Items for Systematic Reviews and Meta-Analyses) guidelines, we searched PubMed, Scopus, and Web of Science for studies published from 2015 to 2025. We included studies involving adults with MDD that used ML models to predict treatment outcomes across 2 or more pharmacological treatments and reported medication-specific prediction outcomes. Risk of bias was assessed using PROBAST-AI (Prediction Model Risk of Bias Assessment Tool for Artificial Intelligence). We conducted a narrative synthesis organized by modeling strategies, data integration approaches, validation methodologies, and performance patterns.

**Results:**

From 5370 initial records, 19 studies met the inclusion criteria, with dataset sample sizes ranging from 49 to 77,226 participants. Studies employed 3 distinct modeling strategies: drug-specific supervised models trained independently for each medication, subtype- or trajectory-based approaches using clustering methods to identify differential response patterns, and a unified differential prediction framework generating calibrated cross-treatment predictions. Performance varied substantially, with area under the curve values ranging from 0.59 to 0.95 and classification accuracies between 62% and 95.4%, though high performance was concentrated in studies with small samples, high-dimensional neurobiological features, and internal-only validation. Only 7 studies conducted external validation, which generally yielded more conservative performance estimates. Feature informativeness was more consistently associated with performance variation than algorithm complexity. Most studies did not formally distinguish between prognostic features predicting general outcomes and predictive features identifying differential medication responses, and none applied formal explainability techniques.

**Conclusions:**

ML for comparative antidepressant selection remains in an early stage of development. Only 1 study implemented a unified framework directly supporting patient-level treatment ranking. Key barriers to clinical translation include insufficient distinction between prognostic and predictive markers, limited cross-trial validation, near-absent calibration reporting, and absent explainability. Future research should prioritize unified comparative frameworks with calibrated predictions, rigorous external validation on diverse cohorts, explicit modeling of heterogeneous treatment effects, and integration of explainability into model development.

## Introduction

Major depressive disorder (MDD) represents one of the most prevalent mental health conditions globally, impacting 1 in 6 adults during their lifetime [1]. The disorder is characterized by persistent depressed mood, anhedonia, and a variety of physical and cognitive symptoms that significantly impair psychosocial functioning and diminish health-related quality of life [2,3]. When the condition becomes severe, patients may exhibit suicidal or self-harming tendencies, increasing the risk of adverse outcomes and health care system burden [4].

Despite the availability of numerous pharmacological interventions, selecting effective antidepressants for individual patients remains challenging due to substantial heterogeneity in symptom presentation, disease progression, and individual treatment response patterns [5,6]. Current antidepressant selection predominantly follows a trial-and-error paradigm, with clinicians initiating treatment based on clinical guidelines and subsequently adjusting medication plans according to observed patient responses [6]. This approach yields modest response rates of approximately 42% to 53%, meaning that nearly half of patients do not achieve adequate symptom improvement, often resulting in prolonged time to remission, poor clinical outcomes, and elevated treatment discontinuation rates [6,7]. These therapeutic inefficiencies also impose significant societal economic costs through increased health care utilization and loss of work productivity [8,9].

The emergence of precision medicine in psychiatry has catalyzed growing interest in leveraging machine learning (ML) methodologies to optimize pharmacotherapy selection for MDD. ML, a subfield of artificial intelligence, encompasses computational methods that enable systems to learn from data and improve performance through experience. ML algorithms can process complex, high-dimensional, and multimodal health care datasets, including electronic health records, genetic markers, neuroimaging data, and clinical assessments, while identifying subtle patterns predictive of treatment outcomes that may not be detected through conventional analytical approaches [10-12]. Recent studies have demonstrated promising results in applying ML to predict treatment outcomes [6,10,11,13-17]. Compared to traditional statistical methods, ML technologies offer enhanced scalability and adaptability, and demonstrate potential for more accurate prediction of individual patient responses to specific therapeutic interventions [18].

The fundamental clinical challenge extends beyond predicting treatment response. Clinical practice inherently involves comparative decision-making. Clinicians must choose which medication to initiate, when to switch to an alternative agent, or whether to augment with additional pharmacotherapy, with each option presenting different mechanisms of action, side effect profiles, and patient-specific considerations [19,20]. A substantial proportion of patients who do not remit on an initial antidepressant achieve remission after switching, suggesting that early nonresponse may reflect suboptimal treatment matching rather than true treatment resistance [21,22]. Prediction models that estimate response to a single medication in isolation cannot directly support this comparative decision-making [23]. Effective treatment selection requires approaches that compare expected outcomes across therapeutic alternatives for individual patients, identifying which specific medication is most likely to provide superior benefit, the essential component for personalized medication selection. In addition, limited model explainability remains a critical barrier to clinical translation. Many ML algorithms function as “black boxes,” providing predictions without transparent reasoning about which patient characteristics drive recommendations [24,25]. For comparative treatment selection, this limitation is particularly concerning because clinicians must understand not only which medication is recommended but also why one option may be preferred over alternatives for a specific patient, enabling them to integrate model outputs with clinical judgment and patient preferences.

In this review, our primary goal is to evaluate the current state of ML approaches that facilitate comparative treatment selection between different medications or medication classes for personalized pharmacotherapy management in MDD. We examine key characteristics of existing models, including their comparative capabilities, data integration approaches, validation methods, and clinical applicability. By systematically evaluating these aspects, this review seeks to identify current limitations and provide insights for developing ML models that can meaningfully guide clinical decision-making in precision psychiatry.

## Methods

### Search Strategy

This systematic review was conducted following the PRISMA (Preferred Reporting Items for Systematic Reviews and Meta-Analyses) guidelines [26]. We performed a search across PubMed, Scopus, and Web of Science databases for all related studies published from 2015 to 2025. The search strategy combined three categories of terms: (1) ML and related approaches, (2) treatment response and outcome prediction, and (3) depression and antidepressant pharmacotherapy, including individual drug classes and specific medication names. The complete search strings for each database are provided in Multimedia Appendix 1.

### Eligibility Criteria

Four independent reviewers screened titles and abstracts, followed by a full-text review of potentially eligible studies. We included original studies involving adult patients diagnosed with MDD that used ML models for predicting treatment outcomes or supporting personalized pharmacotherapy management. Studies were required to examine 2 or more pharmacological treatments or different dosage levels of the same antidepressant, and to report medication-specific prediction outcomes that enable comparison between different antidepressants for individual patients. We excluded studies that examined single treatments without comparison, those focusing only on side effects, nonpharmacological interventions, secondary depression, and those published in languages other than English.

For this review, we defined “comparative treatment selection capability” as a model’s ability to inform individual-level medication choices between different antidepressants. Studies were categorized based on their approaches to handling multiple treatments.

### Data Extraction and Synthesis

Data extraction was conducted independently by reviewers using a standardized extraction form. Discrepancies were reviewed by a third senior reviewer, who made the final decision after discussion with the team. We extracted study information related to study design and ML methodology. Table 1 documents basic study characteristics, including population, study outcome, outcome measurement, and prediction time horizon. Tables 2 and 3 focused on the methodological details of ML, capturing algorithm types, feature selection, validation strategies, and performance metrics. Risk of bias was assessed using the PROBAST-AI (Prediction Model Risk of Bias Assessment Tool for Artificial Intelligence). Two reviewers independently rated each study across 4 domains (participants, predictors, outcome, and analysis), with disagreements resolved through discussion [27,28].

**Table 1.** Experimental design characteristics of the included studies (n=19).

Author	Population (n)	Outcome category	Outcome measure	Prediction horizon (wk)	Intervention
Iniesta et al [29] (2016)	GENDEP^a^ study (793)	Treatment response	HAM-D^b^, MADRS^c^, and BDI^d^ remission	12	SSRI^e^ (escitalopram), TCA^f^ (nortriptyline), combination
Chekroud et al [30] (2017)	STAR*D^g^ + CO-MED^h^ (4706) + duloxetine (2515); Total (7221)	Medication recommendation	HAM-D for 8 wk and QIDS-SR^i^ for 12 wk (symptom clusters)	8‐12	SSRI (escitalopram) + Placebo, SSRI (escitalopram) + NDRI^j^ (bupropion), SNRI^k^ (venlafaxine) + NaSSA^l^ (mirtazapine), SNRI (duloxetine) + SSRIs + Placebo
Crane et al [31] (2017)	University of Michigan (49)	Treatment response	HAM-D (%^m^ change; Hamilton Depression Rating Scale pretreatment to posttreatment)	10	SSRI (escitalopram), SNRI (duloxetine)
Iniesta et al [32] (2018)	GENDEP study (n=430)	Treatment remission	17-item HAM-D^n^	4‐12	SSRI (escitalopram) and SNRI (nortriptyline)
Kautzky et al [33] (2021)	GGRND^o^ study (1070)	Treatment response and remission	HAM-D (treatment response=baseline score >50% and remission <7)	8	SSRI, TCA, antipsychotics, lithium augmentation
Hughes et al [34] (2020)	Site A (51,048), site B (26,176), total (77,226)	Treatment stability	Treatment stability (≥2 prescriptions, ≥30 d apart, ≥90 d duration, MPR ≥80%)	12	SSRI (citalopram, sertraline, fluoxetine, escitalopram, paroxetine), SNRI (venlafaxine, duloxetine), NDRI (bupropion), TCA (nortriptyline, amitriptyline), NaSSA (mirtazapine)
Taliaz et al [6] (2021)	STAR*D (4041)	Treatment response	HAM-D and QIDS^p^ (≥50% reduction)	12‐14	SSRI (citalopram, sertraline), SNRI (venlafaxine)
Athreya et al [35] (2021)	PGRN-AMPS^q^ + ISPC^r^ (947)	Treatment response	HAM-D (treatment response=baseline score >50% and remission total score ≤7)	8	SSRI (citalopram, escitalopram)
Bi et al [36] (2021)	West China Hospital of Sichuan University (610)	Treatment response	HAM-D (treatment response=baseline score >50% and remission total score <8)	6	SSRI (fluoxetine, paroxetine, citalopram, sertraline), SNRI (duloxetine, venlafaxine), TCA (amitriptyline, doxepin, imipramine), NaSSA (mirtazapine)
Nguyen et al [37] (2022)	EMBARC^s^ study (222)	Treatment response	HAM-D (treatment response ≥50% from pre-treatment and remission ≤7)	8‐16	SSRI (sertraline), NDRI (bupropion), placebo
Wang et al [38] (2022)	Multihospital study (430)	Early improvement	20% reduction in 17-item HAMD score	2	SSRIs, SNRIs, combinations, rTMS^t^ + antidepressant, and ECT^u^ + antidepressant
Chen et al [39] (2023)	310	Treatment response	QIDS-SR-16 and HAM-D28	10‐12	SSRI (escitalopram), NDRI (bupropion) or combination (escitalopram + bupropion), SNRI (duloxetine), or placebo
Turner et al [40] (2023)	P64808 and UMCU^v^ (735)	Medication recommendation	Acceptability (prescription duration ≥5 wk) and efficacy (NLP^w^-extracted recovery themes)	14 and 23	SSRI (sertraline, citalopram, escitalopram, fluoxetine, paroxetine, fluvoxamine), SNRI (trazodone, duloxetine, venlafaxine), TCA (amitriptyline, clomipramine, imipramine, doxepin, maprotiline, dosulepine), MAOI^x^ (tranylcypromine, moclobemide, phenelzine), other (bupropion, vortioxetine, agomelatine, hypericum herb, mirtazapine, mianserine)
Fu et al [16] (2024)	COORDINATE-MDD^y^ (1384)	Treatment response	17-item HAM-D	6, 8, or 12	SSRI (sertraline, citalopram, escitalopram), placebo
Curtiss et al [14] (2024)	STAR*D Stage 2 (1439)	Remission	HAM-D (remission = score < 7)	12	SSRI (sertraline, citalopram), SNRI (venlafaxine), NDRI (bupropion), cognitive psychotherapy
Ravan et al [41] (2024)	EMBARC study (224)	Treatment response	17-item HAM-D (treatment response ≥50%)	8	SSRI (sertraline), NDRI (bupropion), placebo
Benrimoh et al [42] (2025)	Pooled antidepressant clinical trials (9042)	Treatment remission	MADRS<11, QIDS-SR-16<6, or HAMD<8	6‐14	10 pharmacological treatments
Carr et al [43] (2025)	GENDEP (714)	Treatment response	HAM-D (remission=score ≤ 7)	12	SSRI (escitalopram), TCA (nortriptyline)
Zhukovsky et al [44] (2025)	EMBARC and Canadian Biomarker Integration Network in Depression-1 (363)	Treatment response	≥50% reduction in depression severity (HDRS or converted MADRS)	8, 16	SSRIs (sertraline and escitalopram)

a GENDEP: Genome-Based Therapeutic Drugs for Depression.

b HAM-D: Hamilton Depression Rating Scale.

c MADRS: Montgomery-Åsberg Depression Rating Scale.

d BDI: Beck Depression Inventory.

e SSRI: selective serotonin reuptake inhibitor.

f TCA: tricyclic antidepressant.

g STAR*D: Sequenced Treatment Alternatives to Relieve Depression.

h CO-MED: combining medications to enhance depression outcomes.

i QIDS-SR: Quick Inventory of Depressive Symptomatology-Self Report version.

j NDRI: norepinephrine-dopamine reuptake inhibitor.

k SNRI: serotonin-norepinephrine reuptake inhibitor.

l NaSSA: noradrenergic and specific serotonergic antidepressant.

m HAM-D%: percent change in HAM-D score.

n HAM-D17: 17-item Hamilton Depression Rating Scale.

o GRND: Genomic-based Response to Depression dataset.

p QIDS: Quick Inventory of Depressive Symptomatology.

q PGRN-AMPS: Pharmacogenomics Research Network-Antidepressant Medication Pharmacogenomics Study.

r ISPC: International SSRI Pharmacogenomics Consortium.

s EMBARC: Establishing Moderators and Biosignatures of Antidepressant Response for Clinical Care.

t rTMS: repetitive transcranial magnetic stimulation.

u ECT: electroconvulsive therapy.

v UMCU: University Medical Center Utrecht.

w NLP: natural language processing.

x MAOI: monoamine oxidase inhibitor.

y COORDINATE-MDD: Coordinated Outcomes in Depression for Individualized and Translational Evaluation in Major Depressive Disorder.

**Table 2.** Feature modality and validation strategy across included studies (n=19).

Feature modality	Reference	Internal validation	External validation
Clinical-only	Iniesta et al [29] (2016) Chekroud et al [30] (2017) Kautzky et al [33] (2021) Hughes et al [34] (2020) Athreya et al [35] (2021) Chen et al [39] (2023) Turner et al [40] (2023) Curtiss et al [14] (2024) Benrimoh et al [42] (2025)	Iniesta et al [29] (2016) Chekroud et al [30] (2017) Kautzky et al [33] (2021) Turner et al [40] (2023) Curtiss et al [14] (2024) Benrimoh et al [42] (2025)	Chekroud et al [30] (2017) Hughes et al [34] (2020) Athreya et al [35] (2021) Chen et al [39] (2023)
Neurobiological-only	Fu et al [16] (2024) Ravan et al [41] (2024)	Fu et al [16] (2024) Ravan et al [41] (2024)	No
Multimodal	Crane et al [31] (2017) Iniesta et al [32] (2018) Taliaz et al [6] (2021) Bi et al [36] (2021) Nguyen et al [37] (2022) Wang et al [38] (2022) Carr et al [43] (2025) Zhukovsky et al [44] (2025)	Iniesta et al [32] (2018) Crane et al [31] (2017) Nguyen et al [37] (2022) Carr et al [43] (2025)	Taliaz et al [6] (2021) Wang et al [38] (2022) Zhukovsky et al [44] (2025)

**Table 3.** Machine learning approaches and predictive performance across included studies (n=19).

Category and reference	Machine learning techniques	Model type	Performance metrics
Drug-specific supervised models			
Iniesta et al [29] (2016)	ENRR^a^	Classification, regression	Explained 5%‐10% variance in symptom improvement; Remission AUC^b^=0.75 for escitalopram and AUC=0.72 for nortriptyline.
Crane et al [31] (2017)	ICA^c^ + multiple regression/RF^d^	Regression	Remission prediction accuracy improved from 74% to 90% after adding fMRI^e^ features.
Iniesta et al [32] (2018)	ENRR	Classification	Remission prediction AUC=0.77 for escitalopram and nortriptyline
Taliaz et al [6] (2021)	SVM^f^ (linear kernel), XGBoost^g^, RF	Classification	Average balanced accuracy of 70.1% across medications in the final test set.
Bi et al [36] (2021)	RF (feature selection) + multiple generalized regression	Classification	Prediction model AUC=77% for SSRI^h^ and 75% for SNRI^i^.
Nguyen et al [37] (2022)	Deep learning (feed-forward neural networks) with data augmentation	Classification, regression	*R*^2^ of 48% for Sertraline and 34% for Bupropion in predicting symptom change.
Ravan et al [41] (2024)	CNN^j^ + symbolic transfer entropy (STE^k^) + ReLORETA^l^	Classification	Overall prediction accuracy >85% (Sertraline: 91%; Placebo: 95.4%; Bupropion: 86.8%).
Curtiss et al [14] (2024)	Super learner ensemble (RF, Elastic Net, NN, XGBoost, etc)	Classification	Prediction AUC=0.51‐0.82 (highest for cognitive therapy at 0.82; Bupropion at 0.70).
Zhukovsky et al [44] (2025)	Elastic net logistic regression and multivariate partial least squares regression (PLS-R)	Classification, regression	Escitalopram AUC=0.66 (balanced accuracy=0.64) Sertraline AUC=0.70 (balanced accuracy=0.71)
Subtype or trajectory-based models			
Chekroud et al [30] (2017)	Hierarchical clustering + linear mixed effects regression + gradient boosting	Regression	Explained variance for symptom clusters: sleep (19.6%), core emotional (14.5%), atypical (15.1%).
Hughes et al [34] (2020)	Prediction-constrained topic modeling + extremely randomized trees/logistic regression	Classification	General stability prediction AUC=0.627‐0.661; drug-specific models performed similarly.
Kautzky et al [33 (2021)	Hierarchical symptom clustering + random forest	Classification	Max prediction accuracy of 0.85 (Cluster IV); SSRI-stratified group accuracy=0.82.
Athreya et al [35] (2021)	GMM^m^ + HMM^n^ + PGM^o^	Classification	Average 8-wk outcome prediction accuracy: SSRI group=77%; Other drugs=72%.
Wang et al [38] (2022)	Hierarchical clustering combined with canonical correlation analysis (CCA) to link functional connectivity to clinical symptoms.	Classification	Achieved an overall cross-dataset accuracy of 72.83%, SSRI accuracy=83.11%, SNRIs accuracy=66.67%
Turner et al [40] (2023)	NLP^p^ + BN^q^	Network analysis	Discovered 28 dependencies between features and outcomes; continuation rates were 66% and 89%
Chen et al [39] (2023)	Hierarchical clustering + penalized logistic regression	Regression	Sleep cluster: *R*^2^=45%, RMSE=81; Atypical cluster: *R*^2^=41%^r^, RMSE^s^=85; Core emotional: *R*^2^=42%, RMSE=92
Fu et al [16] (2024)	Nonlinear semisupervised clustering (HYDRA^t^) + SVM	Classification	Cluster stability ARI^u^=0.61; significant dimension-by-treatment interaction.
Carr et al [43] (2025)	GC^v^ + TDA^w^ + elastic net logistic regression	Classification	Week 4 prediction AUC: escitalopram (0.807), nortriptyline (0.777), combined sample (0.794).
Unified differential prediction model			
Benrimoh et al [42] (2025)	Deep learning	Classification	AUC of 0.65 on the held-out test set. Simulation analysis estimated an increase in population remission rates from 43.63% to 53.98%

a ENRR: elastic net regularized regression.

b AUC: area under the curve.

c ICA: independent component analysis.

d RF: random forest.

e fMRI: functional magnetic resonance imaging.

f SVM: support vector machine.

g XGBoost: eXtreme Gradient Boosting.

h SSRI: Selective Serotonin Reuptake Inhibitor.

i SNRI: Serotonin-Norepinephrine Reuptake Inhibitor.

j CNN: convolutional neural network.

k STE: symbolic transfer entropy.

l ReLORETA: robust exact low-resolution electromagnetic tomography.

m GMM: Gaussian mixture models.

n HMM: hidden Markov model.

o PGM: probabilistic graphical models.

p NLP: natural language processing.

q BN: Bayesian network.

r *R*2: coefficient of determination.

s RMSE: root mean square error.

t HYDRA: heterogeneity through discriminative analysis

u ARI: adjusted rand index (a measure of cluster similarity/stability).

v GC: growth curves.

w TDA: topological data analysis.

We conducted a narrative synthesis, organizing findings by: (1) modeling strategies for handling multiple treatments, (2) data integration approaches, (3) validation methodologies, and (4) performance across different antidepressant classes.

### Risk of Bias Assessment

Risk of bias was assessed using an adaptation of PROBAST incorporating AI-specific signaling questions for ML prediction models [27,28]. The assessment evaluated 4 domains: participants, predictors, outcome, and analysis, with additional considerations for sample size adequacy, high-dimensional predictor handling, class imbalance correction, and overfitting prevention. Separate assessments were conducted for model development and model validation phases. Two reviewers independently rated each study, with disagreements resolved through discussion. All studies were retained regardless of risk of bias classification, given the limited eligible literature. Complete assessment results are reported in Multimedia Appendix 2.

## Results

### Description of the Included Studies

**Figure 1.**
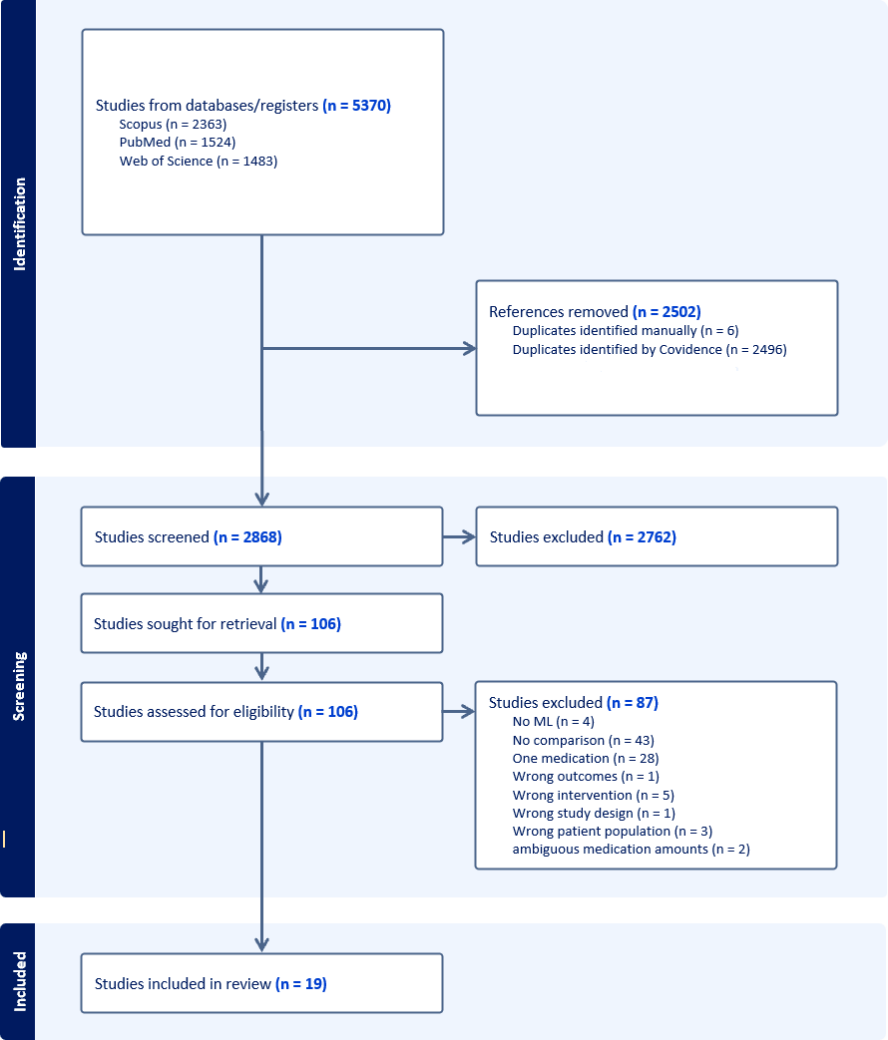
PRISMA (Preferred Reporting Items for Systematic Reviews and Meta-Analyses) flow diagram of study inclusion and exclusion criteria. ML: machine learning.

Our initial search yielded a total of 5370 studies. After removing duplicates and applying inclusion criteria, 19 studies remained (Figure 1).

**Figure 2.**
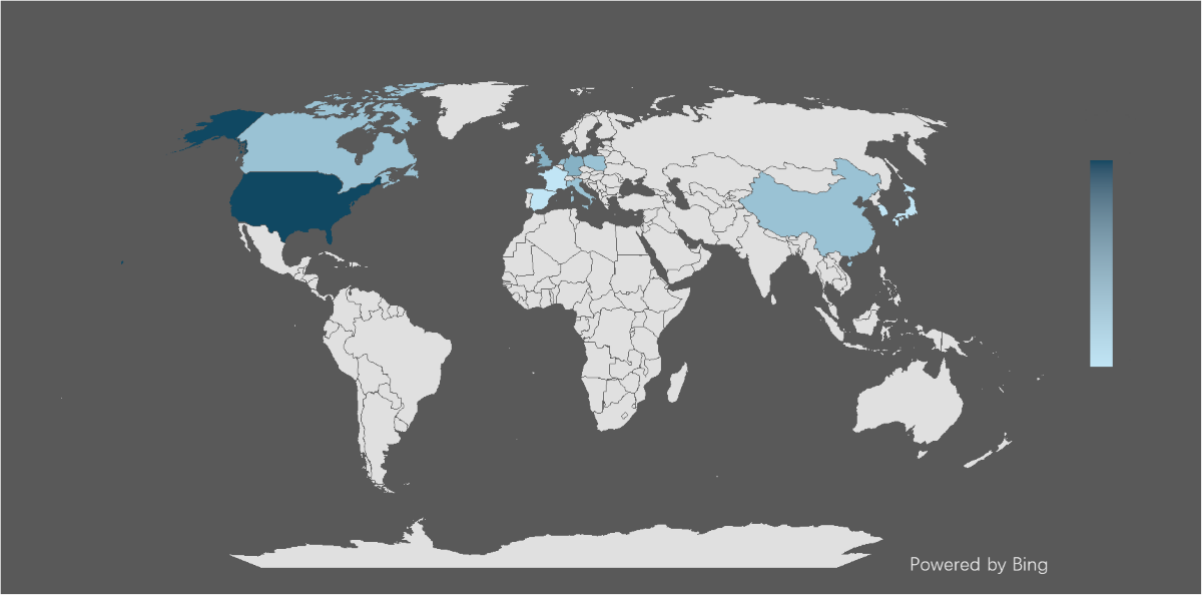
Geographic distribution of datasets used in the included studies (n=19). Figure created in Microsoft Word using the built-in map chart function [45], which is published under limited license per the Microsoft Bing Maps Terms of Use [46].

The 19 included studies demonstrated significant variation in dataset sample sizes for ML model development, ranging from 49 to 77,226 participants. Multiple studies utilized well-established clinical datasets, with considerable overlap across investigations. The Sequenced Treatment Alternatives to Relieve Depression, Genome-Based Therapeutic Drugs for Depression, and Establishing Moderators and Biosignatures of Antidepressant Response for Clinical Care datasets were the most frequently used, with each appearing in three studies [6,14,29,30,32,37,41,43,44]. This indicates significant reuse of established research cohorts. Geographically, the majority of studies originated from Western countries, particularly the United States and European institutions, while 2 investigations were conducted in China [36,38]. This distribution reflects the current concentration of ML research infrastructure in established academic medical centers (Figure 2).

### Data Input, Feature Modality, and Validation Strategy

Most studies relied on structured clinical and demographic data, including symptom ratings, treatment history, and comorbidities [14,29,30,33-35,39,40,42]. Neurobiological features were examined in 2 studies, using pretreatment electroencephalogram connectivity and structural magnetic resonance imaging, respectively [16,41]. The remaining 8 studies pursued multimodal integration, combining neurobiological or genetic data with clinical variables [6,31,32,36-38,43,44]. Turner et al [40] introduced natural language processing methods to derive structured predictors from unstructured clinical notes, highlighting an alternative pathway for utilizing real-world data.

Internal validation was the predominant approach across studies. Only 7 studies conducted external validation on independent datasets or cross-site samples, though the scope varied considerably [6,30,34,35,38,39,44]. Five studies validated their full modeling framework on independent cohorts, while Zhukovsky et al [44] conducted cross-trial validation between 2 randomized controlled trials (RCTs). Taliaz et al [6] conducted external validation only on a single medication model. Among these externally validated studies, 4 relied on clinical-only features, suggesting that external validation has been predominantly pursued in studies using routinely available data. This pattern, alongside the tendency for neurobiological studies to use smaller cohorts with internal validation only, precludes direct performance comparisons across feature modality categories.

**Figure 3.**
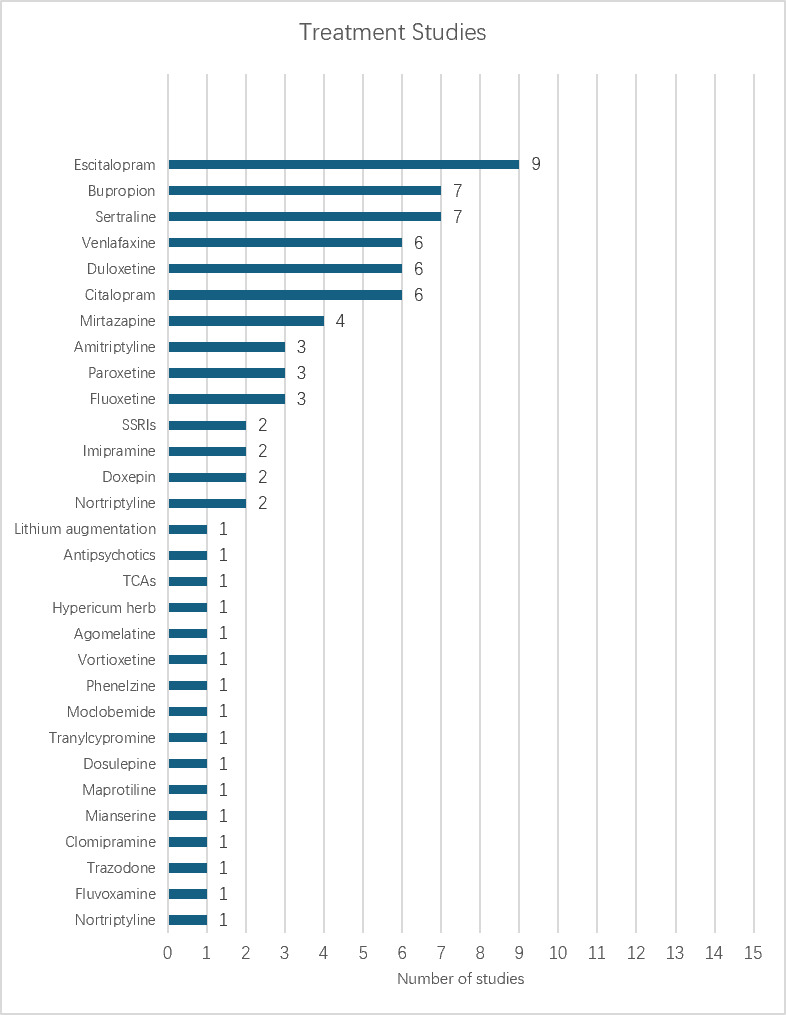
Distribution of antidepressant treatments modeled across included studies. SSRI: Selective Serotonin Reuptake Inhibitor; TCA: tricyclic antidepressant.

Antidepressant coverage was also uneven (Figure 3). Escitalopram and bupropion were most frequently modeled, followed by sertraline, venlafaxine, duloxetine, and citalopram. A small number of studies additionally examined treatment classes (eg, Selective Serotonin Reuptake Inhibitors [SSRIs], tricyclic antidepressants) or augmentation strategies (eg, lithium).

### Explainability and Interpretability

Most of the included studies did not apply formal explainable artificial intelligence techniques, except for Benrimoh et al [42] and Nguyen et al [37]. Benrimoh et al [42] used gradient-based saliency maps using the GuidedBackprop algorithm to generate patient-level interpretability reports, providing clinicians with the top 5 features driving each individual treatment prediction. Nguyen et al [37] used the partial derivative method to rank feature importance learned by their deep learning models, reporting the 20 most important predictive features for each treatment arm. Explainable artificial intelligence techniques are designed to provide transparent and interpretable explanations of ML model predictions, helping users understand how models arrive at specific decisions [47]. Beyond these 2 studies, model interpretability was either implicitly embedded within model architecture or addressed through basic post hoc statistical descriptions. Several studies employed inherently interpretable models, including logistic regression, Bayesian networks, or penalized linear models with coefficient inspection [29,32,39,40,43,44]. In many cases, these models were selected not explicitly for interpretability, but because they enabled effective feature selection for predicting antidepressant treatment response. Similarly, symptom-based clustering was employed to define patient subgroups with distinct response patterns, providing an indirect rationale for treatment matching [16,30,38,39].

### Outcome Measures

The included studies demonstrated considerable variation in outcome measurement approaches. Depression severity was assessed using standardized rating scales in 17 of 19 (89.5%) studies. The Hamilton Depression Rating Scale was the most utilized measure across studies in various forms, including the 17-item and 28-item versions. Within this group, 3 studies combined the Hamilton Depression Rating Scale with the Quick Inventory of Depressive Symptomatology Self-Report, and 1 study additionally incorporated both the Montgomery-Åsberg Depression Rating Scale and the Beck Depression Inventory. The remaining 2 studies adopted alternative outcome measures. Hughes et al [34] evaluated treatment stability, and Turner et al [40] assessed treatment acceptability and efficacy.

Despite this general reliance on standardized scales, the definitions of treatment response varied substantially. Most studies defined response as a reduction of 50% or more in depressive symptoms on a selected scale, while some required a reduction of more than 50%. Wang et al [38] adopted a lower threshold of a reduction of 20% or more for defining early improvement at 2 weeks. Remission was typically characterized as achieving scores below predetermined scale-specific cutoff points, most commonly 7 or fewer.

The predictive time horizons, the periods over which outcomes are predicted, ranged from 2 weeks to 163 days. Most predicted time horizons were from 6 to 12 weeks. Only 1 study extended to 163 days for longer-term outcome assessment [40].

### ML Techniques

A wide array of ML techniques has been employed across the included studies to predict antidepressant treatment outcomes (Table 3). Linear and logistic regression methods were frequently applied, including elastic net regression, which is valued for its feature selection and interpretability [29-32,34,39,43,44]. Tree-based methods, such as random forest and gradient boosting, were implemented in both stand-alone and ensemble configurations [30,33,34,36]. Support vector machines and deep learning models were less common. Deep learning was used in studies that incorporated high-dimensional neuroimaging or multimodal inputs, but was also applied to low-dimensional clinical and demographic data at scale [6,37,41,42]. Two studies used probabilistic graphical or network-based approaches to model relationships between patient features and treatment outcomes [35,40].

Across studies, the choice of algorithm broadly corresponded to the dimensionality of input data. Deep learning and multivariate regression were employed in studies with high-dimensional neuroimaging inputs [37,41,44]. Linear and penalized regression models were applied in studies using structured clinical variables, and tree-based or ensemble methods appeared in studies combining multiple data types [6,29,30,34,36,39].

In addition, the studies included varied in how they handled multiple pharmacological interventions. Several studies trained independent models for each antidepressant arm, applying the same algorithm and feature set separately, which enabled drug-specific predictions but did not support direct comparisons across treatments [6,14,29,31,32,36,37,41,44]. The second group of studies employed clustering or trajectory-based methods, grouping patients into subtypes or modeling treatment paths and then examining differential response patterns [30,33-35,38-40,43]. The third approach, represented by Benrimoh et al [42], developed a unified differential treatment prediction framework in which a single model simultaneously generated calibrated remission probabilities across 10 pharmacological treatments for each individual patient, enabling direct cross-treatment comparison. The training sample size also differed across approaches. Drug-specific models often had to rely on smaller effective cohorts per arm, and clustering approaches required sufficiently large subgroups to ensure stable results.

### Performance

**Figure 4.**
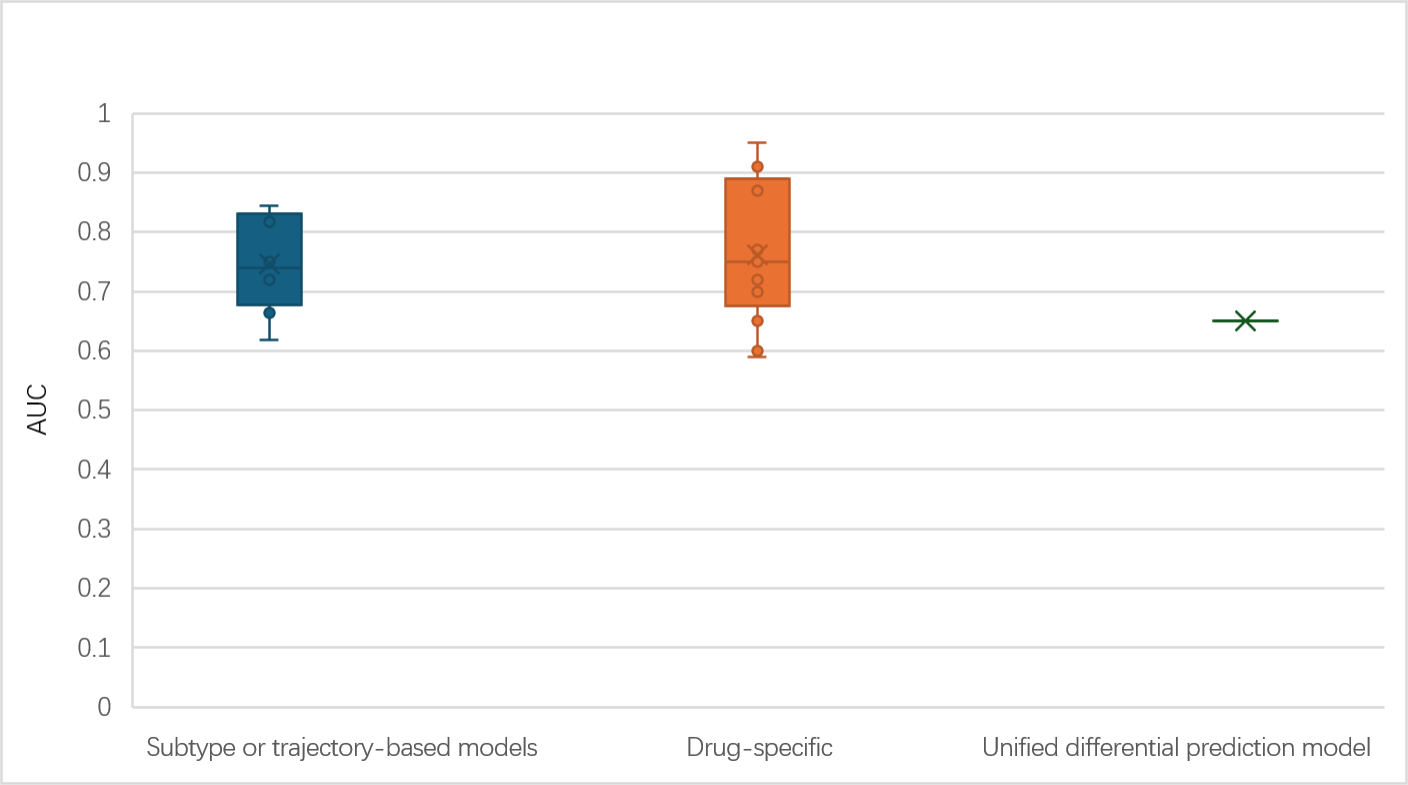
Comparison of area under the curve (AUC) across drug-specific supervised models, subtype- or trajectory-based models, and unified differential prediction model.

Reported performance metrics varied by medication arm, modeling strategy, and outcome type (Table 3). Studies demonstrated substantial heterogeneity in model performance, with area under the curve (AUC) values ranging from 0.59 to 0.95 for classification tasks and classification accuracies between 62% and 95.4% across conditions (Figure 4). However, such high performance levels were uncommon and concentrated in a small number of studies, while most reported more moderate predictive accuracy.

At the structural level, subtype- or trajectory-based models that did not distinguish between medications generally achieved lower but more stable performance compared with drug-specific approaches, which demonstrated greater heterogeneity but included some of the highest reported values. For example, Hughes et al [34] reported AUC values of 0.627 to 0.661 for general stability prediction across external validation sites, whereas among drug-specific models, Ravan et al [41] achieved classification accuracies above 85% using electroencephalogram-based deep learning with internal validation only. This contrast illustrates the characteristic trade-off between generalizability and peak performance observed across the 2 approaches.

Incorporating early treatment response data as additional predictive features demonstrated consistent advantages across multiple studies. Carr et al [43] reported AUC values increasing from 0.703 at baseline to 0.794 at week 4 and 0.844 at week 6 in the combined sample. Similarly, Zhukovsky et al [44] found that replacing pretreatment depression severity with week 2 scores improved cross-trial AUC from 0.58 to 0.68 for the clinical-only model and up to 0.79 for the clinical plus neuroimaging model.

Four studies used regression frameworks to predict changes in continuous symptom severity [30,31,39,44]. Chen et al [39] applied penalized regression within symptom-based clusters, reporting *R*² values of up to 45% in the sleep cluster, with root mean square error values ranging from 81 to 92. Replicating the approach of Chekroud et al [30], these results contrasted with the lower values observed in the original models, where *R*² was below 0.20 [39]. Zhukovsky et al [44] used multivariate partial least squares regression incorporating functional connectivity features, reporting predicted-vs-observed correlations of 0.31 to 0.39 in cross-trial validation. Turner et al [40] used a Bayesian network approach to model probabilistic dependencies among patient features and treatment outcomes. Although not directly comparable to classification or regression models, their approach identified stable network structures and reported a 6% to 11% gain in treatment selection for specific subgroups. However, traditional performance metrics such as area under the receiver operating characteristic curve or root mean square error were not reported, which limits comparability.

Among the 3 modeling strategies, Benrimoh et al [42] reported the only unified differential prediction framework, achieving an AUC of 0.65 on the held-out test set across 10 pharmacological treatments. While this AUC was lower than many drug-specific models, the model’s simulation analysis estimated an increase in population remission rates from 43.15% to 53.99% when treatment was selected based on model recommendations rather than random assignment.

Examining performance patterns across studies, several factors appeared systematically associated with variation in reported model performance. Feature complexity and data modality represented the most consistent source of performance differences. Studies incorporating neurobiological inputs, such as neuroimaging, electrophysiological signals, or genetic markers, generally reported higher predictive performance than those relying exclusively on clinical and demographic variables [31,41,43,44]. By comparison, studies using only structured clinical and demographic inputs reported more modest classification performance [14,29,30,33-35,40,42].

However, this feature-performance association covaried with sample size and validation approach in ways that complicate straightforward interpretation. Studies employing neurobiological features tended to use smaller research cohorts and relied predominantly on internal cross-validation. Conversely, the largest study in the review utilized structured clinical data and conducted external validation across independent clinical sites yet reported comparatively lower AUC values. Among the 7 externally validated studies, the reported performance was generally more conservative [6,30,34,35,38,39,44]. Taliaz et al [6] achieved an average balanced accuracy of 70.1% internally, but the only model validated externally yielded 61.3%. Zhukovsky et al [44] similarly demonstrated a performance gap, with the best pretreatment cross-trial AUC of 0.62 to 0.67 for SSRI generalization across 2 independent RCTs. However, Chen et al [39] maintained consistent performance, with *R*² values of 41% to 45% on independent RCT datasets, suggesting that external validation does not uniformly attenuate results when the modeling approach is well aligned to the clinical context. Model architecture, by contrast, appeared to play a comparatively secondary role. Both complex approaches, such as convolutional neural networks, and simpler regularized regression methods achieved strong performance when paired with informative neurobiological features, while ensemble methods applied to clinical-only data did not consistently outperform simpler algorithms [41,43]. This observed pattern indicates that variation in reported performance across studies corresponded more closely to input feature characteristics than to algorithm type.

### Risk of Bias Assessment

Risk of bias was assessed separately for model development and validation phases (Multimedia Appendix 2). For model development, the overall risk of bias was low in 10 studies, high in 4 studies, and unclear in 4 studies (Tables S1 and S2 in Multimedia Appendix 2). The participants and predictors domains demonstrated consistently low risk, while the outcome and analysis domains raised more frequent concerns. Common methodological limitations included incomplete reporting of missing data handling, absence of calibration assessment, and insufficient detail regarding overfitting prevention. For model validation, 9 studies were rated as low risk, 5 as high risk, and 5 as unclear. Applicability concerns were low across all studies.

## Discussion

### Principal Findings

Our review identified only 19 eligible studies out of 5370 initially screened, demonstrating a critical need for research that applies ML for comparative modeling across multiple antidepressant medications. This scarcity represents a critical gap because most studies focused on only a single pharmacological treatment or grouped treatment arms [36,48-57]. These approaches cannot inform the clinician’s central task of selecting between therapeutic options for an individual patient. This field remains constrained by methodological inconsistencies, limited generalizability, and a lack of explainability, which collectively hinder translation into routine clinical practice.

Beyond the scarcity of comparative studies, the uneven distribution of antidepressants examined raises questions about model applicability. Current models are optimized for predicting outcomes among the most commonly prescribed medications. Yet, clinicians often face decisions involving less frequently studied agents, particularly when managing treatment-resistant depression or addressing specific patient contraindications [42]. The scarcity of studies examining tricyclic antidepressants, monoamine oxidase inhibitors, or atypical antidepressants means that ML-guided treatment selection may be least available precisely when clinical decision-making is most complex. Moreover, most studies fail to address combination therapy. Our review found that only 1 study explicitly included patients receiving augmentation therapy and treated all medication combinations as a single category, without distinguishing between specific drug-drug interactions or predicting optimal combination strategies [33]. Modeling each medication separately, or treating all combinations as equivalent, fails to account for the complex pharmacological interactions inherent in combination therapy [58].

Among the included studies, 3 distinct strategies for individualized treatment selection were identified. The first strategy was to train separate models for each antidepressant, which allows for tailored prediction per medication but lacks a unified comparative framework, making it difficult to rank or recommend treatments across options for a single patient. Because each model is trained independently, observed differences in predictive performance may reflect sample variation, modeling noise, or hyperparameter settings, rather than true pharmacological differences. These models, therefore, function primarily as treatment-specific response prediction tools rather than direct treatment recommendation systems.

The second strategy was to cluster patients based on symptom profiles or other baseline characteristics and then assess treatment response within each subgroup [16,30,33,35,39]. These studies aimed to improve treatment matching by identifying homogeneous patient subtypes that might respond differentially to treatment. However, as the number of interventions assessed increases, creating meaningful clusters becomes increasingly difficult due to the exponential growth in possible treatment-subgroup combinations [59]. Additionally, as cluster numbers increase, interpreting which patient characteristics drive differential treatment responses becomes more complex and less clinically actionable [59,60]. This approach relies on unsupervised techniques and post hoc comparisons. It requires large sample sizes to ensure stable clustering solutions. External validation is also needed to confirm that identified subgroups replicate across different populations [61-63]. More generally, this approach supports response stratification rather than comparative treatment selection.

The third strategy focuses on developing a unified differential treatment prediction framework that simultaneously generates calibrated remission probabilities across multiple pharmacological treatments for the same individual patient [42]. This approach directly addresses the comparative selection challenge by producing patient-level treatment rankings rather than relying on post hoc comparisons of separately trained models. Notably, only 1 study implements such a framework, suggesting a need for further investigation [42]. The emergence of this third strategy marks a conceptual shift from “Can we predict response?” toward “Which treatment should this patient receive?” This question most closely mirrors clinical decision-making.

Several studies integrated multimodal data, including neuroimaging, genetic markers, and electroencephalogram signals, generally demonstrating improved performance over clinical-only models [14,29,35,43]. However, this advantage should be interpreted cautiously. Studies incorporating multimodal features simultaneously increased total feature dimensionality, and without systematic ablation studies, it is difficult to disentangle modality-specific signals from the effect of higher data volume [64,65]. This ambiguity is compounded by confounding between data modality, sample size, and validation approach. Neurobiological studies tended to use smaller cohorts with internal validation only, whereas larger studies using clinical data with external validation reported more conservative performance [16,31,37,41]. Practical barriers further limit multimodal integration, as neuroimaging and genetic testing require dedicated infrastructure and personnel, which constrain scalability. Future work should evaluate whether causal inference frameworks designed for heterogeneous treatment effects improve selection accuracy relative to conventional predictive models.

Beyond data modality considerations, a fundamental limitation underlying these strategies is the insufficient distinction between prognostic and predictive features. Prognostic features predict overall treatment outcome regardless of medication choice, whereas predictive features identify differential responses through treatment-by-covariate interactions [66,67]. Only predictive features can directly inform comparative medication selection, but most included studies did not formally distinguish between these 2 types of markers. Among drug-specific modeling studies, differences in model outputs or performance across treatment arms may reflect sampling variability or modeling noise rather than genuine pharmacological specificity [6,14,36,37,41]. Similarly, studies employing clustering, general prediction models, or other frameworks primarily identified prognostic patterns, with post hoc treatment comparisons generating hypotheses rather than confirming predictive effects [16,29-31,33-35,39,40,43]. Two studies from the same research group provided the most direct empirical evidence on this distinction. Iniesta et al [29] demonstrated through cross-drug validation that models trained on escitalopram patients explained negligible variance in nortriptyline outcomes and vice versa. Additionally, in their other study, Iniesta et al [32] extended this finding with a larger genetic feature set, confirming drug-specific prediction with a validation AUC of 0.77 for both drugs, while cross-drug AUC fell to a chance level between 0.57 and 0.62. However, Zhukovsky et al [44] demonstrated that models trained on sertraline data in one trial could predict escitalopram response in an independent trial at above-chance levels, suggesting that some predictive signal may generalize across SSRIs with similar mechanisms of action. This finding complicates the prognostic-predictive distinction, as it raises the possibility that within-class cross-drug generalizability reflects shared pharmacological pathways rather than purely prognostic features. Future research should prioritize frameworks that explicitly model heterogeneous treatment effects and adopt cross-drug validation to differentiate predictive from prognostic signals.

Another major barrier relates to inconsistencies in outcome definitions and evaluation metrics. Variations in response thresholds and remission criteria can reclassify patients at the margin, potentially influencing model training [68]. Combined with inconsistent evaluation metrics and varying prediction horizons, this heterogeneity hinders reliable cross-study comparison and makes quantitative meta-analysis impossible [69]. To advance the field, consensus development regarding standardized evaluation frameworks will be important.

The near absence of formal explainability techniques across all included studies represents a significant barrier to clinical translation. In psychiatry, explainability is essential for building clinician trust, ensuring ethical accountability, and supporting treatment selection decisions [47,70,71]. A noted barrier to the adoption of ML tools by clinicians relates to predictions without clear explanations, particularly in high-stakes scenarios like antidepressant selection [72]. Future studies should embed explainability into the model development pipeline and collaborate with mental health professionals to ensure clinical relevance.

Dataset redundancy and limited generalizability raise additional concerns. Repeated use of the same datasets introduces risks of overfitting to cohort-specific patterns and model tuning based on familiar distributions, potentially inflating performance estimates [69,73]. The highest reported performances share a common methodological profile. Small sample sizes combined with high-dimensional neurobiological features and internal-only validation, a pattern consistent with overfitting [41]. The performance gap between internal and external validation further supports this concern. For example, Taliaz et al [6] achieved 70.1% balanced accuracy internally but only 61.3% on externally validated models. In addition, Zhukovsky et al [44] demonstrated that models showed reduced performance when applied across independent trials compared to within-trial settings. Moreover, nearly all studies reported only discrimination metrics such as AUC, while calibration, which assesses whether predicted probabilities accurately reflect observed outcomes, was almost entirely absent. Only Benrimoh et al [42] reported calibrated remission probabilities, demonstrating that this approach is feasible within a unified differential prediction framework. For treatment selection, well-calibrated probability estimates are arguably more important than discrimination, as clinicians need reliable absolute risk estimates to compare treatment alternatives [74-76]. Limited geographic and demographic diversity compounds these concerns, as genetic variation and cultural differences in symptom expression may cause models to over-rely on region-specific features [36,77-80]. The predominance of internal validation, sometimes using overlapping cohorts as ostensibly independent test sets, further limits confidence in reported performance. Future studies should prioritize external validation on prospective, multisite, demographically diverse cohorts and report calibration alongside discrimination metrics.

Finally, few studies have addressed long-term outcomes. Most predictions have targeted short-term responses within 6 to 12 weeks, with very limited attention given to sustained remission, relapse prevention, or longitudinal treatment trajectories. However, prediction horizons inherently differ in difficulty. Longer-term forecasting is more challenging due to increased variability, patient dropout, and cumulative confounders [81]. Moreover, prediction horizons correspond to distinct clinical applications. Short-term response prediction can guide early treatment adjustments, while long-term modeling is crucial for evaluating real-world effectiveness and preventing relapses [82]. Yet these long-term outcomes are central to real-world decision-making and should be prioritized in future modeling efforts.

### Limitations

We acknowledge the limitations in our review. First, our search was restricted to 3 databases and English-language publications, potentially missing relevant studies in other databases, languages, or gray literature sources. Second, we may have missed relevant studies due to variations in indexing terminology across the interdisciplinary field of ML and psychiatry, where different vocabularies are used to describe similar comparative treatment prediction approaches. Third, although the risk of bias was assessed using PROBAST-AI, all 19 studies were retained regardless of their bias classification due to the limited number of eligible studies. Findings from studies rated as high risk should therefore be interpreted with caution. Fourth, the heterogeneity in study designs, methodologies, and reporting standards across included studies prevented quantitative meta-analysis, limiting our review to a narrative synthesis and reducing our ability to provide pooled estimates of model performance. Finally, the confounding between data modality, sample size, and validation approach across included studies prevents us from isolating the independent contribution of multimodal features to model performance, and future ablation studies are needed to clarify whether observed performance advantages reflect modality-specific information or greater overall data volume.

### Conclusion

This systematic review reveals that ML for comparative antidepressant selection remains in an early stage of development. Three distinct modeling strategies were identified, which are drug-specific models, subtype- or trajectory-based approaches, and a unified differential prediction framework. Yet only the last directly supports patient-level treatment ranking, and it was implemented in a single study. Most studies did not formally distinguish between prognostic and predictive features, limiting the capacity to identify which patients benefit differentially from specific medications. Additional barriers include limited cross-trial validation, near-absent calibration reporting, and the lack of formal explainability techniques. To advance toward clinical translation, future research should prioritize unified comparative frameworks with calibrated predictions, rigorous external validation on diverse cohorts, explicit modeling of heterogeneous treatment effects, and integration of explainability into model development. Only by addressing these fundamental gaps can ML become a trustworthy and clinically valuable tool for optimizing antidepressant selection in MDD.

## Supplementary material

10.2196/89352 Multimedia Appendix 1 Search strategy.

10.2196/89352 Multimedia Appendix 2 Risk of bias assessment.

10.2196/89352 Checklist 1 PRISMA checklist.

## Abbreviations

AUC: area under the curve
MDD: major depressive disorder
ML: machine learning
PRISMA: Preferred Reporting Items for Systematic Reviews and Meta-Analyses
PROBAST-AI: Prediction Model Risk of Bias Assessment Tool for Artificial Intelligence
RCT: randomized controlled trial
SSRI: Selective Serotonin Reuptake Inhibitor
