# Supplementary material for: Machine Learning for Comparative Antidepressant Selection in Major Depressive Disorder: Systematic Review
Source: JMIR Ment Health. 2026 May 13;13:e89352. doi: 10.2196/89352 (PMC13170422; doi:10.2196/89352)
Supplement: Multimedia Appendix 1 [file mental-v13-e89352-s001.docx]

Multimedia Appendix

Multimedia Appendix 1 Search Strategy-----------------------------------1

## Multimedia Appendix 1: Search Strategy

**Pubmed**: ("artificial intelligence" OR ai OR "deep learning" OR "machine learning" OR "ML" OR "neural network" OR" personalized medicine" OR "personalized treatment" OR "algorithmic prescribing" OR "individualized treatment" )AND (trajector* OR "treatment response" OR "response to treatment" OR "treatment selection" OR "comparative effectiveness" OR "treatment effect") AND ("depression" OR "mdd" OR "antidepressant*" OR "depressive" OR "anxiety" OR "PTSD" OR "ADHD" OR "SSRI" OR "SNRI" OR "MAOI" OR "TCAs" OR "NDRI" OR "NaSSA" OR "SMS" OR "SARI" OR "selective serotonin reuptake inhibitor*" OR "serotonin and norepinephrine reuptake inhibitor*" OR "norepinephrine–dopamine reuptake inhibitor" OR "noradrenergic and specific serotonergic antidepressant" OR "NMDA receptor antagonists" OR "serotonin antagonist and reuptake inhibitors" OR "serotonin modulator and stimulator") Filters: Full text, English, Exclude preprints, from 2015/1/1 - 2025/1/1

**Web of Science**: ("artificial intelligence" OR ai OR "deep learning" OR "machine learning" OR “ML” OR "neural network" OR" personalized medicine" OR "personalized treatment" OR "algorithmic prescribing" OR “individualized treatment” )AND (trajector* OR "treatment response" OR "response to treatment" OR "treatment selection" OR "comparative effectiveness" OR "treatment effect") AND ("depression" OR "mdd" OR "antidepressant*" OR "depressive" OR “anxiety” OR “PTSD” OR “ADHD” OR "SSRI" OR "SNRI" OR "MAOI" OR "TCAs" OR “NDRI” OR “NaSSA” OR “SMS” OR “SARI” OR “selective serotonin reuptake inhibitor*" OR "serotonin and norepinephrine reuptake inhibitor*" OR “norepinephrine–dopamine reuptake inhibitor” OR “noradrenergic and specific serotonergic antidepressant” OR “NMDA receptor antagonists” OR “serotonin antagonist and reuptake inhibitors” OR “serotonin modulator and stimulator”) (Topic) and Meeting or Letter or Data Paper or Retracted Publication or Early Access or Editorial Material or Meeting Abstract or Book Chapters or Proceeding Paper or Review Article (Exclude – Document Types) and English (Languages)

**Scopus**: TITLE-ABS-KEY(("artificial intelligence" OR ai OR "deep learning" OR "machine learning" OR "ML" OR "neural network" OR" personalized medicine" OR "personalized treatment" OR "algorithmic prescribing" OR "individualized treatment" )AND (trajector* OR "treatment response" OR "response to treatment" OR "treatment selection" OR "comparative effectiveness" OR "treatment effect") AND ("depression" OR "mdd" OR "antidepressant*" OR "depressive" OR "anxiety" OR "PTSD" OR "ADHD" OR "SSRI" OR "SNRI" OR "MAOI" OR "TCAs" OR "NDRI" OR "NaSSA" OR "SMS" OR "SARI" OR "selective serotonin reuptake inhibitor*" OR "serotonin and norepinephrine reuptake inhibitor*" OR "norepinephrine–dopamine reuptake inhibitor" OR "noradrenergic and specific serotonergic antidepressant" OR "NMDA receptor antagonists" OR "serotonin antagonist and reuptake inhibitors" OR "serotonin modulator and stimulator")) AND PUBYEAR > 2014 AND PUBYEAR < 2026 AND ( EXCLUDE ( DOCTYPE,"bk" ) OR EXCLUDE ( DOCTYPE,"er" ) OR EXCLUDE ( DOCTYPE,"dp" ) OR EXCLUDE ( DOCTYPE,"sh" ) OR EXCLUDE ( DOCTYPE,"tb" ) OR EXCLUDE ( DOCTYPE,"le" ) OR EXCLUDE ( DOCTYPE,"cr" ) OR EXCLUDE ( DOCTYPE,"no" ) OR EXCLUDE ( DOCTYPE,"cp" ) OR EXCLUDE ( DOCTYPE,"ed" ) OR EXCLUDE ( DOCTYPE,"ch" ) OR EXCLUDE ( DOCTYPE,"re" ) ) AND ( LIMIT-TO ( LANGUAGE,"English" ) )
