# Supplementary material for: Machine Learning for Comparative Antidepressant Selection in Major Depressive Disorder: Systematic Review
Source: JMIR Ment Health. 2026 May 13;13:e89352. doi: 10.2196/89352 (PMC13170422; doi:10.2196/89352)
Supplement: Multimedia Appendix 2 [file mental-v13-e89352-s002.docx]

Multimedia Appendix

Multimedia Appendix 2 Risk of Bias Assessment------------------------2

## Multimedia Appendix 2

Supplementary Table 1: PROBAST-AI Risk of Bias Assessment for Model Development

| Question | Iniesta  (2016) | Chekroud  (2017) | Crane (2017) | Iniesta (2018) | Hughes (2020) | Athreya (2021) | Bi (2021) | Kautzky (2021) | Taliaz (2021) | Nguyen (2022) | Wang (2022) | Chen (2023) | Turner (2023) | Curtiss (2024) | Fu (2024) | Ravan (2024) | Benrimoh (2025) | Carr (2025) | Zhukovsky (2025) |
| --- | --- | --- | --- | --- | --- | --- | --- | --- | --- | --- | --- | --- | --- | --- | --- | --- | --- | --- | --- |
| 1.1 Were appropriate data sources used (e.g., cohort, RCT, or nested case–control study data)? | Y | Y | Y | Y | Y | Y | Y | Y | Y | Y | Y | N/A | Y | Y | Y | Y | Y | Y | Y |
| 1.2 Was an appropriate study design used? | Y | Y | Y | Y | Y | Y | Y | Y | Y | Y | Y | N/A | Y | Y | Y | Y | Y | Y | Y |
| 1.3 Were all inclusions and exclusions of participants appropriate and result in a representative dataset? | Y | Y | Y | Y | Y | Y | Y | Y | Y | Y | Y | N/A | Y | Y | Y | Y | Y | PY | Y |
| Final rating domain 1 (risk of bias) | L | L | L | L | L | L | L | L | L | L | L | N/A | L | L | L | L | L | L | L |
| Applicability | L | L | L | L | L | L | L | L | L | L | L | N/A | L | L | L | L | L | L | L |
| 2.1 Were predictors defined and assessed in a similar way for all participants? | Y | Y | Y | Y | Y | Y | Y | Y | PY | Y | Y | N/A | Y | Y | PY | Y | Y | PY | Y |
| 2.2 Was any pre-processing of predictors similar for all participants? | Y | Y | Y | Y | Y | Y | Y | Y | Y | Y | Y | N/A | Y | NI | PY | Y | Y | Y | Y |
| 2.3 Were predictor assessments made without knowledge of outcome data? | Y | Y | Y | Y | Y | Y | Y | Y | Y | Y | Y | N/A | Y | Y | Y | Y | Y | PY | Y |
| 2.4 Were the predictors included in the model available at the time the model was intended to be used? | Y | Y | Y | Y | Y | Y | Y | Y | Y | Y | Y | N/A | Y | Y | Y | Y | Y | Y | Y |
| Final rating domain 2 (risk of bias) | L | L | L | L | L | L | U | L | L | L | L | N/A | L | U | U | L | L | U | L |
| Applicability | L | L | L | L | L | L | L | L | L | L | L | N/A | L | L | L | L | L | L | L |
| 3.1 Was the outcome determined appropriately? | Y | Y | Y | Y | PY | Y | Y | Y | Y | Y | Y | N/A | Y | Y | Y | Y | Y | Y | Y |
| 3.2 Were outcomes defined and assessed in a similar way for all participants? | Y | Y | Y | Y | Y | Y | Y | PY | Y | Y | Y | N/A | Y | Y | Y | Y | U | Y | Y |
| 3.3 Were outcome assessments made without use of predictor data? | Y | PY | PY | Y | PY | Y | Y | U | Y | Y | Y | N/A | Y | Y | Y | Y | Y | Y | Y |
| 3.4 Was the time interval between predictor assessment and outcome assessment appropriate? | Y | Y | Y | Y | Y | Y | Y | Y | Y | Y | Y | N/A | Y | Y | Y | Y | Y | Y | Y |
| Final rating domain 3 (risk of bias) | L | L | H | L | L | L | L | H | L | L | L | N/A | U | L | L | L | H | L | L |
| Applicability | L | L | L | L | U | L | L | L | L | L | L | N/A | L | L | L | L | L | L | L |
| 4.1 Was there evidence that the sample size was reasonable? | Y | PY | PY | Y | PY | Y | Y | PY | Y | Y | Y | N/A | Y | Y | Y | PY | Y | PY | PY |
| 4.2 Were continuous and categorical predictors handled appropriately? | Y | Y | Y | Y | Y | Y | Y | U | Y | Y | Y | N/A | Y | NI | Y | Y | Y | Y | Y |
| 4.3 Were participants with missing or censored data handled appropriately in the analysis? | PY | PY | PY | Y | U | Y | U | U | Y | U | Y | N/A | Y | PY | NI | PY | PY | Y | PY |
| 4.4 If methods to address class imbalance were used, were the model or the model predictions recalibrated? | N/A | N/A | N/A | N/A | N/A | N/A | U | N/A | N/A | Y | Y | N/A | U | N/A | N/A | N/A | N/A | N/A | N/A |
| 4.5 Were methods used to address potential model overfitting? | Y | Y | PY | Y | PY | Y | Y | PY | Y | Y | Y | N/A | Y | Y | Y | Y | Y | Y | Y |
| Final rating domain 4 (risk of bias) | U | U | L | L | U | U | H | H | U | U | Y | N/A | U | U | U | U | L | L | L |
| Final rating total (risk of bias) | L | L | H | L | L | L | H | H | L | U | L | N/A | U | U | U | L | H | L | L |

Supplementary Table 2: PROBAST-AI Risk of Bias Assessment for Model Validation

| Question | Iniesta  (2016) | Chekroud  (2017) | Crane (2017) | Iniesta (2018) | Hughes (2020) | Athreya (2021) | Bi (2021) | Kautzky (2021) | Taliaz (2021) | Nguyen (2022) | Wang (2022) | Chen (2023) | Turner (2023) | Curtiss (2024) | Fu (2024) | Ravan (2024) | Benrimoh (2025) | Carr (2025) | Zhukovsky (2025) |
| --- | --- | --- | --- | --- | --- | --- | --- | --- | --- | --- | --- | --- | --- | --- | --- | --- | --- | --- | --- |
| 1.1 Were appropriate data sources used (e.g., cohort, RCT, or nested case–control study data)? | Y | Y | Y | Y | Y | Y | Y | Y | Y | Y | Y | Y | Y | Y | Y | Y | Y | Y | Y |
| 1.2 Was an appropriate study design used? | Y | Y | Y | Y | Y | Y | Y | Y | Y | Y | Y | Y | Y | Y | Y | Y | Y | Y | Y |
| 1.3 Were all inclusions and exclusions of participants appropriate and result in a representative dataset? | Y | Y | Y | Y | Y | Y | Y | Y | Y | Y | Y | Y | Y | Y | Y | Y | Y | PY | Y |
| Final rating domain 1 (risk of bias) | L | L | L | L | L | L | L | L | L | L | L | L | L | L | L | L | L | L | L |
| Applicability | L | L | L | L | L | L | L | L | L | L | L | L | L | L | L | L | L | L | L |
| 2.1 Were predictors defined and assessed in a similar way for all participants? | Y | Y | Y | Y | Y | Y | Y | Y | PY | Y | Y | Y | Y | Y | PY | Y | Y | PY | Y |
| 2.2 Was any pre-processing of predictors similar for all participants? | Y | Y | Y | Y | Y | Y | Y | Y | Y | Y | Y | Y | Y | NI | PY | Y | Y | Y | Y |
| 2.3 Were predictor assessments made without knowledge of outcome data? | Y | Y | PY | Y | Y | Y | Y | Y | Y | Y | Y | Y | Y | Y | Y | Y | Y | PY | Y |
| 2.4 Were the predictors included in the model available at the time the model was intended to be used? | Y | Y | Y | Y | Y | Y | Y | Y | Y | Y | Y | Y | Y | Y | Y | Y | Y | Y | Y |
| Final rating domain 2 (risk of bias) | L | L | L | L | L | L | U | L | L | L | L | L | L | U | U | L | L | U | L |
| Applicability | L | L | L | L | L | L | L | L | L | L | L | L | L | L | L | L | L | L | L |
| 3.1 Was the outcome determined appropriately? | Y | Y | Y | Y | PY | Y | Y | Y | Y | Y | Y | N | Y | Y | PY | Y | Y | Y | Y |
| 3.2 Were outcomes defined and assessed in a similar way for all participants? | Y | Y | Y | Y | Y | Y | Y | PY | Y | Y | Y | U | Y | Y | Y | Y | N | Y | Y |
| 3.3 Were outcome assessments made without use of predictor data? | Y | PY | PN | Y | Y | Y | Y | U | Y | Y | Y | Y | Y | Y | Y | Y | Y | Y | Y |
| 3.4 Was the time interval between predictor assessment and outcome assessment appropriate? | Y | Y | Y | Y | Y | Y | Y | Y | Y | Y | Y | U | Y | Y | Y | Y | P | Y | Y |
| Final rating domain 3 (risk of bias) | L | L | H | L | L | L | L | H | L | L | L | H | U | L | L | L | H | L | L |
| Applicability | L | L | L | L | U | L | L | L | L | L | L | L | L | L | L | L | L | L | L |
| 4.1 Was model evaluation based on only apparent performance avoided? | Y | Y | Y | Y | Y | Y | U | Y | Y | Y | Y | Y | Y | Y | Y | Y | Y | Y | Y |
| 4.2 Was there evidence that the sample size was reasonable? | N/A | N/A | N/A | Y | N/A | N/A | Y | N/A | N/A | N/A | Y | Y | N/A | N/A | N/A | N/A | Y | N/A | N/A |
| 4.3 Were participants with missing or censored data handled appropriately in the analysis? | N/A | N/A | N/A | Y | N/A | N/A | U | N/A | N/A | N/A | Y | U | N/A | N/A | N/A | N/A | N/A | N/A | N/A |
| 4.4 If methods to address class imbalance were used, was the evaluation done in a dataset without imbalance correction? | N/A | N/A | N/A | Y | N/A | N/A | U | N/A | N/A | N/A | Y | N/A | N/A | N/A | N/A | N/A | N/A | N/A | N/A |
| 4.5 If data splitting was done to create training and test datasets, was there evidence that data leakage was avoided? | N/A | N/A | N/A | Y | N/A | N/A | U | N/A | N/A | N/A | Y | Y | N/A | N/A | N/A | N/A | N/A | N/A | N/A |
| 4.6 If resampling methods were used to evaluate model performance, were all model development steps replicated in the resampling process? | N/A | N/A | N/A | Y | N/A | N/A | U | N/A | N/A | N/A | Y | Y | N/A | N/A | N/A | N/A | N/A | N/A | N/A |
| 4.7 Was the predictive performance of the model evaluated appropriately, e.g.,  calibration, discrimination, and net benefit? | N/A | N/A | N/A | Y | N/A | N/A | Y | N/A | N/A | N/A | Y | Y | N/A | N/A | N/A | N/A | N/A | N/A | N/A |
| Final rating domain 4 (risk of bias) | U | U | L | L | U | L | H | H | L | U | Y | U | U | U | U | U | L | L | L |
| Final rating total (risk of bias) | L | L | H | L | U | L | H | H | L | U | L | H | U | U | U | L | H | L | L |
